# Broad Ligament Leiomyomata - An Important Differential Diagnosis in Huge Abdominal Mass: A Case Report

**DOI:** 10.31729/jnma.8908

**Published:** 2025-03-31

**Authors:** Amogh Bahadur Basnyat, Ganesh Dangal, Aruna Karki, Hema K Pradhan, Ranjana Shrestha, Kabin Bhattachan

**Affiliations:** 1 Department of Obstetrics and Gynaecology, Kathmandu Model Hospital Institute of Health Sciences, Bagbazar, Kathmandu

**Keywords:** *leiomyoma*, *extra-uterine*, *broad ligament*

## Abstract

Leiomyomas are a common gynaecological condition affecting 20-30% of women over the age of 35, with prevalence decreasing following menopause. Leiomyomas are most commonly found within the uterus. Rarer extra-uterine locations include the broad ligament, cervix, and vagina. We present an unusual case of multiple Left Broad Ligament Leiomyomata that extended from Left Iliac Fossa and Hypogastrium to Epigastric region in a 50-year-old female. Our case highlights the importance for extra-uterine leiomyoma to be considered as a differential diagnosis in patients presenting with huge abdominal mass suspicious of adnexal tumor or malignancy.

## INTRODUCTION

Leiomyomas (fibroids) are benign smooth-muscle neoplasms that typically originate from the myometrium. They are usually firm, well circumscribed, and localized to the pelvic cavity. Extrauterine leiomyomas (EULs) are rare, and their etiology is unclear. Their locations include the broad ligament, cervix, and vagina.^1^ Uncommon manifestations of leiomyoma include intravenous leiomyomatosis, parasitic leiomyoma, and benign metastasizing leiomyoma.^2^ We are describing an unusual case of multiple Left Broad Ligament Leiomyomata that extended from Left Iliac Fossa and Hypogastrium to Epigastric region and the largest one of which weighed 3.9 kgs. It was surgically removed successfully.

## Case

A 50-year-old P2L2 presented to OPD with complaint of abdominal distention for the last 6 months. She was apparently well prior to that period when she started noticing her abdomen getting progressively distended. It was uncomfortable and somewhat painful to her, especially on prone position. She didn't have any history of fever, loss of weight or appetite, night sweats, change in bowel or bladder habits. She didn't report any bleeding per rectum or per vaginam. Her menstrual cycles were reported as regular with average pain and flow. She was a P2 with both her deliveries being normal vaginal ones. She didn't have significant past medical or surgical history. On examination, her general condition was fair and she wasn't icteric or pale. On abdominal exam, she had a mass of about 32 weeks size; it was non-tender with restricted mobility and firm to hard consistency. On per vaginal exam, her uterus couldn't be appreciated separate from the mass and there was bilateral forniceal fullness.

Her USG abdomen and pelvis revealed a huge, heterogenous echogenic mass arising from pelvis, cephalad up to upper abdomen just underneath the xiphisternum. Its size was 16.5x7.7x12.1 cm. There was no definite increase in vascularity. Small amount of free fluid was noted at lower anterior abdomen, of depth 3.4 cm. Normal uterus and ovaries were not identified. Bilateral kidneys showed no hydronephrosis.

Her MRI abdomen and pelvis revealed an approximately 11.4 AP x 22.6 TR x 27.1 CC size moderate heterogenous enhancing thin wall capsulated lobulated outline solid mass in pelvi-abdominal region with mass effect. Uterus was displaced anterosuperiorly and to the right side. Urinary bladder was compressed and displaced inferiorly. Findings were reported as highly suspicious left adnexal mass, most likely sex cord stromal tumor or fibrothecomas.

**Figure 1:**
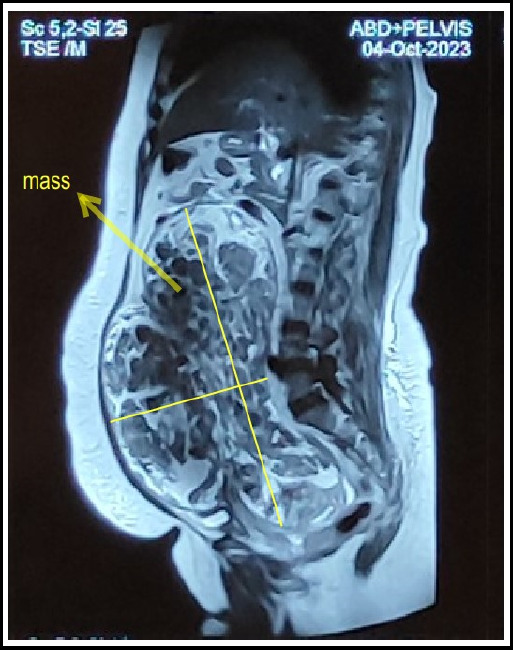
MRI Sagittal view of abdomen-pelvis showing thin walled, capsulated lobulated solid mass.

**Figure 2:**
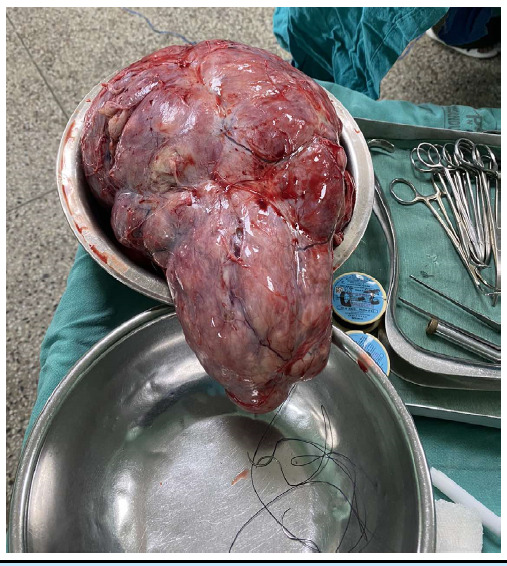
Leiomyomata

Her tumor markers including CA125, CEA, CA 19.9, AFP and CEA came within normal limits.

Hence, she was planned for Laparotomy and proceed. Intraoperatively, she was found to have left broad ligament multiple fibroids, with the largest one measuring 30 x 25 cm and weighing 3.9 kgs. The whole mass was multiloculated and extended from left iliac fossa and hypogastrium to the epigastric region. Cut section of the mass showed multicystic areas with whorled pattern with yellowish, viscous fluid contained in between the lobes. Bilateral tubes and ovaries were normal along with the uterus. Along with the excision of the entire mass, total abdominal hysterectomy with bilateral salpingo-oophorectomy with omental and para-aortic lymph node sampling was also done and the specimens sent for histopathological examination.

Report of the histopathology confirmed the surgical diagnosis of benign Leiomyomata.

**Figure 3:**
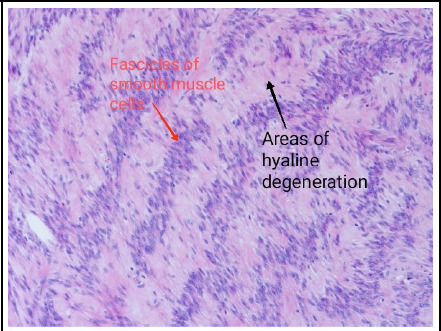
Fascicles and interlacing bundles of smooth muscle cells with extensive areas of hyaline degeneration seen in the histopathology of the mass (H & E stain, x 100)

## DISCUSSION

Leiomyomas (fibroids) are a common gynaecological condition and they affect about 20-30% of women over the age of 35.^2^ Their prevalence is found to decrease after menopause. Leiomyomas are found most commonly within the uterus. However, there are rarer extra-uterine locations that include the broad ligament, cervix, and vagina. Uncommon manifestations of leiomyomas also include intravenous leiomyomatosis, parasitic leiomyoma, and benign metastasizing leiomyoma.^2^

Uterine leiomyomas are benign monoclonal tumours of the myometrium consisting of smooth muscle cells, fibroblasts, and extracellular matrix. They are encapsulated by a pseudo-capsule formed from areolar tissue and muscle fibres separating the tumour from the local surrounding structures. The growth of leiomyomas is thought to be dependent on vascularization, hormones, and patient age.^2^

Their clinical features also vary depending on their size and locations.^1^ Common presenting symptoms of uterine leiomyomas include heavy menstrual bleeding, dysmenorrhoea, and pelvic discomfort. More unusual presenting symptoms include thrombosis, haematometra, haemoperitoneum, and lower urinary tract symptoms.^2^ However, in our case no such symptoms were encountered, given the extrauterine location of the mass. Extra-uterine leiomyomas usually present with pain and secondary complications from their compression effects as a result of their location, as this was evident in our case.^1^

The most common extra uterine site is said to be broad ligament with an incidence of <1%.^1^ There are two types of broad ligament fibroids; false broad ligament fibroid that grows into the broad ligament from the uterus, and true broad ligament fibroid that arises from sub-peritoneal connective tissue of the ligament.^1^ In our case, it was found to be a true broad ligament fibroid.

Extra-uterine leiomyoma EUL can be among the differential diagnosis of uterine leiomyoma along with others being broad ligament fibroid, solid ovarian mass, pedunculated subserosal fibroid or lymphadenopathy.^1^ Clinicians should have a high suspicion of index of EUL as some diagnostic modalities can miss these, as we saw in our case. In a case report by Dulewad et al, the authors presented a case of EUL mistaken as pregnancy. Jha et al also state in their report that it can be difficult to make diagnosis using ultrasonography especially when the fibroid is big and displaces adjacent structures away.^3,4^ Similar diagnostic dilemma was apparent in the radiological reports in our case too, where it was diagnosed as most suspicious of sex cord stromal tumor or fibrothecoma.

Leiomyomas can be incidentally found during routine physical examination. They are investigated quickest by Ultrasound which can detail their size, site, and number. Magnetic resonance imaging MRI is utilized to evaluate the leiomyoma in relation to the rest of the pelvis, including relations to neurovascular structures and nearby organs, such as in our case. MRI has the highest sensitivity and can describe secondary changes like degenerations, calcifications, or necrosis.^1^ The use of MRI as in our case can assist in the planning of operative management. However, imaging methods are not reliable in determining the probability of malignant transformation which is reserved for histological evaluation.^2^

Following symptomatic treatment, uterine artery embolization, myomectomy, and hysterectomy are used in the treatment of symptomatic leiomyoma.^2^ Surgery is the mainstay for the management of symptomatic leiomyomas. Due to the size and location, sometimes surgery is challenging due to the proximity of nearby pelvic and abdominal structures.^1^

Laparoscopic surgery can be an option, but surgeons should note that the fibroid can be much larger than the initial appearance because the pelvis and retroperitoneal space can accommodate as a reservoir and needs careful hemostasis during dissection as can be very vascularized and in close relations to nearby vital structures (ureters, internal iliacs, and nerves).^5^

## CONCLUSION

Broad ligament fibroids are unusual pelvic tumors that can cause difficulties in diagnosis as they mimic malignant tumors; their surgical management can also be difficult due to their locations and sizes. Clinicians should think of extra-uterine leiomyomas as a differential diagnosis of adnexal masses. Management can be by surgery but can pose complications; hence, surgical expertise is required for successful outcome.
